# Prognostic factors and treatment outcomes of allogeneic stem cell transplantation in lymphoid malignancy

**DOI:** 10.1007/s44313-025-00060-y

**Published:** 2025-02-10

**Authors:** Hyungsoon Kim, Haerim Chung, Hye Won Kook, Soo-Jeong Kim, Yu Ri Kim, Hyunsoo Cho, June-Won Cheong

**Affiliations:** 1 Blood Cancer Research Institute, Yonsei University College of Medicine, Seoul, 03722 Republic of Korea; 2 Division of Hematology, Department of Internal Medicine, Severance Hospital, Yonsei University College of Medicine, 50-1 Yonsei-Ro, Seodaemun-Gu, Seoul, 03722 Republic of Korea

**Keywords:** Allogeneic stem cell transplantation, Lymphoid malignancies, Prognostic factors

## Abstract

**Purpose:**

Allogeneic stem cell transplantation (allo-SCT) is a potentially curative treatment option for patients with relapsed or refractory lymphoid malignancies. However, the prognostic factors influencing survival outcomes in these patients remain poorly defined. This study aimed to evaluate the clinical variables associated with progression-free survival (PFS) and overall survival (OS) in patients undergoing allo-SCT for lymphoid malignancies.

**Methods:**

We analyzed 58 patients who underwent allo-SCT for lymphoid malignancies, including B-cell lymphoma (BCL, *n* = 20), Hodgkin lymphoma (*n* = 3), multiple myeloma (*n* = 9), natural killer/T-cell lymphoma (NK/TCL, *n* = 4), and T-cell lymphoma (TCL, *n* = 22). Clinical factors such as HLA matching and post-transplant response status were assessed for their association with survival outcomes using univariate and multivariate analyses.

**Results:**

The median PFS and OS were 27.4 months and 30.6 months, respectively. In univariate analysis, both HLA matching and complete remission (CR) status after transplantation were associated with superior PFS and OS. However, multivariate analysis identified only post-transplant CR as an independent predictor of improved survival. Subgroup analysis revealed that HLA matching was significantly associated with better PFS in patients with BCL and NK/TCL, and with better OS only in BCL. Post-transplant CR was consistently associated with improved PFS and OS across BCL, NK/TCL, and TCL subtypes.

**Conclusion:**

Post-transplant response is a key prognostic factor influencing survival in allo-SCT for lymphoid malignancies. Achieving complete remission after transplantation may help guide post-SCT management and risk-adapted therapeutic strategies.

**Supplementary Information:**

The online version contains supplementary material available at 10.1007/s44313-025-00060-y.

## Introduction

Patients with relapsed or refractory (R/R) lymphoid malignancies have poor outcomes, with limited treatment options available after failure of immunotherapies, including chimeric antigen receptor (CAR) T-cells, bispecific antibodies, and antibody-drug conjugates [1]. Although innovative, these therapies are hindered by target antigen loss, limited CAR T-cell persistence, and an immunosuppressive tumor microenvironment. Given these limitations, allogeneic stem cell transplantation (allo-SCT) is a potential alternative, particularly for patients who relapse early after autologous stem cell transplantation (ASCT) or those with high-risk features, such as refractoriness to immunochemotherapy [2]. Unlike ASCT, allo-SCT prevents contamination with lymphoma cells in reinfused hematopoietic stem cells and harnesses the graft-versus-lymphoma (GVL) effect to eliminate residual disease [3, 4]. However, this procedure carries significant risks, including transplant-related mortality and graft-versus-host disease (GVHD), with non-relapse mortality (NRM) rates as high as 19.2% [5–8]. Despite these risks, advances in patient management and donor selection have reduced NRM, establishing allo-SCT as a viable option [9].

Allo-SCT is one of the treatment options for managing lymphoid malignancies, offering durable progression-free survival (PFS) in patients with mature T-cell lymphomas (TCLs) and improved survival in those with natural killer (NK)/TCL [10, 11]. The procedure has also demonstrated particular efficacy in young, chemotherapy-sensitive patients with multiple myeloma (MM) [12]. However, the factors influencing allo-SCT success, particularly those related to recipient and donor characteristics, remain inadequately explored. Notable factors included sex, age, human leukocyte antigen (HLA) matching, conditioning regimen, and pre-transplant status [13–15]. For instance, recipient sex has been identified as a significant prognostic factor, while donor sex primarily affects female recipients. Age at transplantation also plays a crucial role, with benefits observed in various age groups. Although HLA matching has been traditionally emphasized, recent studies suggest that haploidentical donors may provide outcomes comparable to those of fully HLA-matched donors. The choice of conditioning regimen, whether myeloablative or reduced intensity, remains a subject of ongoing debate [16, 17]. Additionally, patients with well-controlled disease tend to have better post-transplant prognoses [10, 18].

Despite these advancements, the specific prognostic factors influencing allo-SCT success in lymphoid malignancies remain poorly understood. Therefore, this study aimed to evaluate the prognostic factors and treatment outcomes associated with allo-SCT in lymphoid malignancies to refine eligibility criteria that can improve patient selection and outcomes.

## Patients and methods

### Patients’ ethics approval and consent to participate

This study was approved by the Institutional Review Board of Severance Hospital, Seoul, Republic of Korea (IRB No. 4-2021-1597) and was conducted in accordance with the ethical guidelines of the Declaration of Helsinki. All patients provided written informed consent before participating in the study. A total of 58 patients diagnosed with lymphoid malignancies who underwent allo-SCT at Severance Hospital between 2000 and 2023 were included in this study. Pre-transplant treatment of lymphoid malignancy subtypes is presented in Supplementary Table 1. Stem cells were mobilized using granulocyte colony-stimulating factor, and patients received conditioning regimens with combinations of fludarabine, melphalan, anti-thymocyte globulin, busulfan, cyclophosphamide, and total body irradiation, tailored according to the disease type and donor source. The pre-transplant conditioning protocols are demonstrated in Supplementary Table 2. Patient follow-up continued until December 2023.

### Definition

The conditioning regimen intensity was categorized as myeloablative conditioning (MAC) or reduced-intensity conditioning (RIC). Complete remission (CR) was defined as the total disappearance of all detectable clinical evidence of disease. Partial remission (PR) was defined as the regression of measurable disease without the emergence of new lesions, whereas refractory or relapsed disease was classified as progressive disease (PD). Pre-transplant status was defined as the disease status within one week before transplantation, while post-transplant status referred to the condition one week after transplantation. Additionally, PFS was defined as the time from transplantation to death from any cause. Overall survival (OS) was assessed from transplantation to death from any cause. NRM was defined as death occurring without prior lymphoma relapse. Acute and chronic GVHD were graded according to established protocols.

### Statistical analysis

Patients were divided into subgroups according to the cancer type, including B-cell lymphoma (BCL), Hodgkin’s disease (HD), MM, NK/TCL, and TCL. Survival analyses were conducted for both the entire cohort and the five subgroups. Additionally, analyses were performed by combining HD with BCL (BCL + HD) and NK/TCL with TCL (TCL + NK/TCL). The Kaplan–Meier method was used to calculate OS and PFS probabilities. To analyze the incidence of clinical events, patients were categorized into three subgroups: (1) BCL + HD, (2) MM, and (3) TCL + NK/TCL. Cumulative incidence curves were applied to calculate the incidence of NRM, relapse, acute, and chronic GVHD. The Cox proportional hazards regression model was employed for both the univariate and multivariate analyses. Factors included in the analyses were: lymphoma subtype, age at transplantation, sex, donor relationship, conditioning regimen, transplantation date, HLA match, pre-/post-transplant status, and the presence of acute or chronic GVHD. Statistical significance was set at *P* < 0.05. Results were presented as hazard ratios (HR) with 95% confidence intervals (95% CI). Only factors significantly associated with PFS or OS in univariate analysis were included in the multivariate analysis. Additionally, survival analyses were conducted for variables significantly associated with survival using the Kaplan–Meier method for the entire cohort, as well as for the BCL and TCL subgroups. A Sankey diagram was used to demonstrate changes in treatment status before and after transplantation. All analyses were performed using IBM SPSS version 26.0.

## Results

### Baseline patient characteristics

**Fig. 1:**
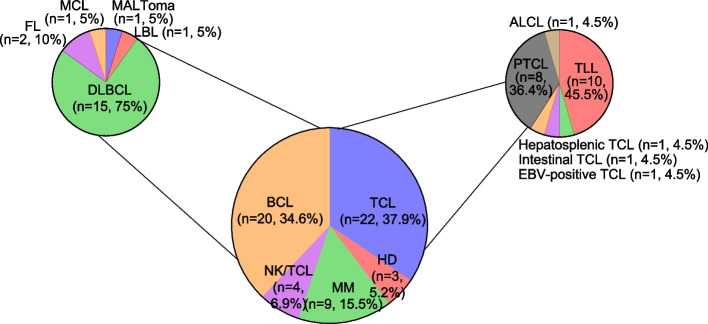
Distribution of lymphoid malignancies in allogeneic stem cell transplantation recipients. MALToma, mucosa-associated lymphoid tissue lymphoma; MCL, mantle cell lymphoma; DLBCL, diffuse large B-cell lymphoma; FL, follicular lymphoma; LBL, B-cell lymphoblastic lymphoma; TLL, T-lymphoblastic lymphoma; TCL, T-cell lymphoma; PTCL, peripheral T-cell lymphoma; ALCL, anaplastic large cell lymphoma

A total of 58 patients with relapsed or refractory lymphoid malignancies underwent allo-SCT. Baseline characteristics are summarized in Supplementary Table 3. Among these patients, 20 (34.9%) had BCL, 22 (37.9%) had TCL, four (6.9%) had NK/TCL, three (5.2%) had HD, and nine (15.5%) had MM (Fig. 1). Most patients (83%; *n* = 48) underwent RIC, whereas 17% (*n* = 10) underwent MAC. Transplantation was performed between 2000 and 2023, with 29% (*n* = 17) between 2000–2009, 59% (*n* = 34) between 2010–2019, and 12% (*n* = 7) between 2020–2023. HLA matching was achieved in 41% (*n* = 24) of cases, whereas 34% (*n* = 20) of patients received transplants from haploidentical donors. None of the patients received CAR-T before or after allo-SCT. At the time of transplantation, 19% (*n* = 11) of the patients achieved CR, meanwhile, 41% (*n* = 24) achieved CR post-transplantation. Before transplantation, 17% (*n* = 10) of patients had PR and 48% (*n* = 28) had PD. After transplantation, 3% (*n* = 2) of the patients remained in PR and 43% (*n* = 25) continued to have PD. Additionally, 29.3% (*n* = 17) of the patients developed acute GVHD and 10.3% (*n* = 6) experienced chronic GVHD. The characteristics of the patients with GVHD are summarized in Supplementary Table 4.

Among patients with BCL, 75% (*n* = 15) were identified with diffuse large B-cell lymphoma (DLBCL) (Fig. 1). Additionally, two cases of follicular lymphoma and one case each of mucosa-associated lymphoid tissue lymphoma (MALToma), mantle cell lymphoma (MCL), and lymphoblastic lymphoma (LBL) were observed (Fig. 1). Among TCLs, peripheral T-cell lymphoma (PTCL) and T-LBL (TLBL) were the most common, accounting for 36.4% (*n* = 8) and 45.5% (*n* = 10) of cases, respectively (Fig. 1). The remaining patients had hepatosplenic TCL, intestinal TCL, Epstein-Barr virus-positive TCL, or anaplastic large cell lymphoma (ALCL).

**Fig. 2:**
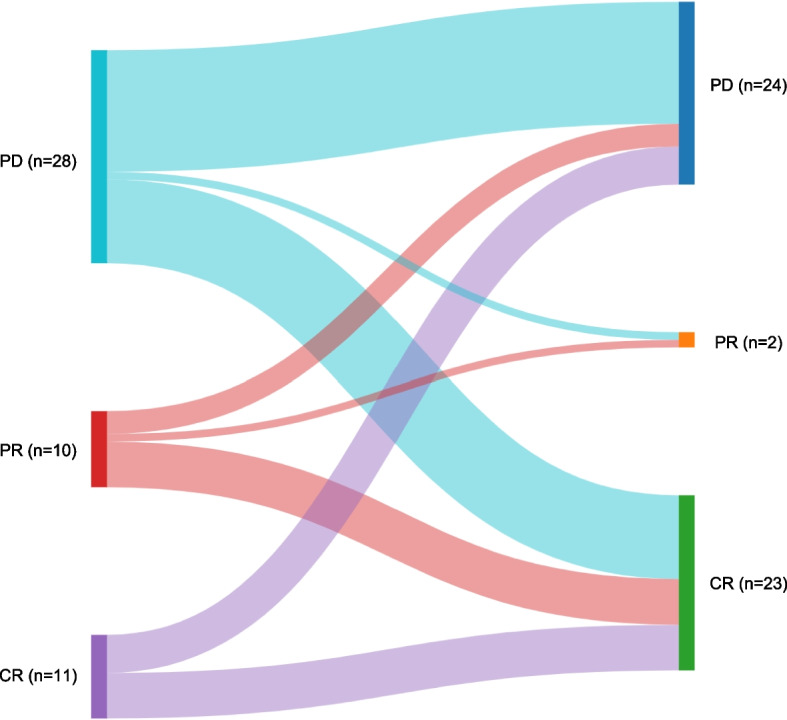
Disease status before and after allogeneic stem cell transplantation

Sixteen patients with PD maintained PD status after transplantation (Fig. 2). One patient demonstrated a status change from PD to PR and 11 patients with PD before transplantation achieved CR (Fig. 2). Among the 10 recipients with pre-transplant PR, six achieved CR, one achieved PR, and three remained with PD after transplantation (Fig. 2). Six patients who achieved CR before transplantation managed to maintain this status, while five progressed from CR before transplantation to PD status after receiving allo-SCT (Fig. 2).

### Survival outcomes

**Fig. 3:**
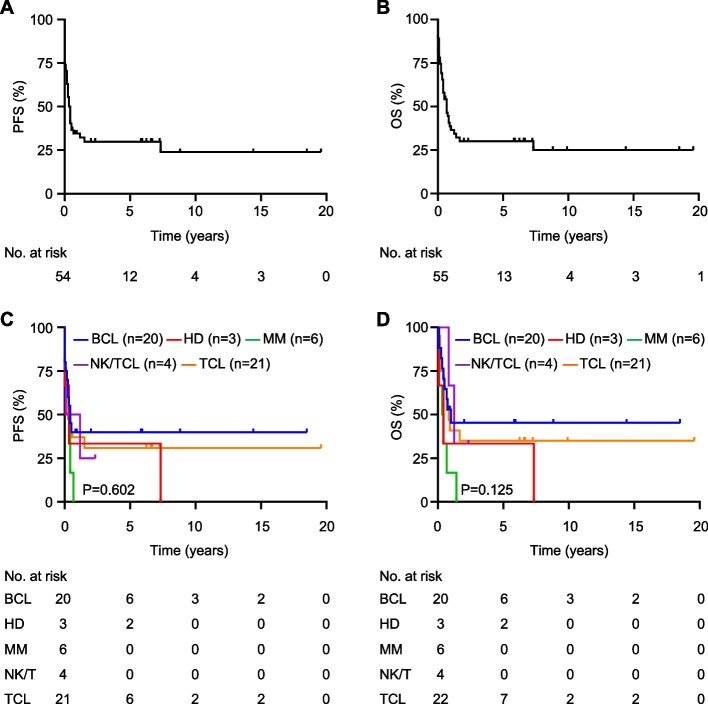
Survival outcomes in allogeneic stem cell transplantation recipients with lymphoid malignancies. **A**, **B** Progression-free survival (PFS) (*n* = 58) and overall survival (OS) (*n* = 58) of all patients. **C**, **D** PFS and OS according to the subtype of lymphoid malignancies. *P*-values were determined by log-rank test

At the time of analysis, 65.5% (*n* = 38) of the patients had died, whereas 31.0% (*n* = 18) remained alive. The median PFS was 27.4 months, and the median OS was 30.6 months (Fig. 3A, B). Two years post-allo-SCT, the PFS and OS rates were 29.9% and 30.1%, respectively (Fig. 3A, B). No significant differences were observed in PFS or OS among the various lymphoma subtypes (Fig. 3C, D). The median PFS for BCL, HD, MM, NK/TCL, and TCL was 35.8, 30.7, 6.0, 10.8, and 29.0 months, respectively (Fig. 3C, D). Median OS values were 36.8 months (BCL), 31.3 months (HD), 6.0 months (MM), 13.3 months (NK/TCL), and 34.8 months (TCL) (Fig. 3C, D). Two-year PFS rates were 40.0%, 33.3 %, 25.0 %, and 30.9% for BCL, HD, NK/TCL, and TCL, respectively (Fig. 3C, D). None of the patients with MM survived beyond 5 years (Fig. 3C, D).

Survival analysis, which classified HD as BCL and NK/TCL as TCL, also revealed no significant differences in PFS or OS (Supplementary Fig. 1). In this analysis, the median PFS was 35.1 months for BCL and 26.0 months for TCL, with a median OS of 36.1 months in patients with BCL and 31.5 months in those with TCL (Supplementary Fig. 1). The 2-year PFS rates were 39.1% and 29.5% for BCL and TCL, respectively.

**Fig. 4:**
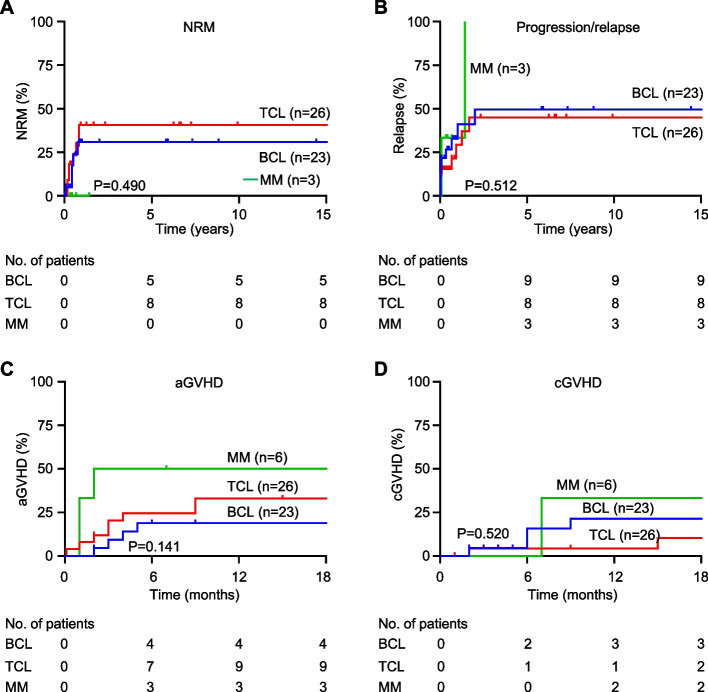
Incidence of non-relapse mortality (NRM), mortality, and graft-versus-host disease (GVHD) in allogeneic stem cell transplantation recipients for lymphoid malignancies. **A** Cumulative incidence of NRM in subgroups according to the lymphoid malignancy subtype. **B** Cumulative incidence of progression/relapse in subgroups. **C**, **D** Cumulative incidence of acute and chronic GVHD in subgroups

The 5-year incidence of NRM was 21.7% and 30.8% in the BCL and TCL subgroups, respectively (Fig. 4A, B). None of the patients with MM died of NRM. The relapse rates in patients with BCL, TCL, and MM were 39.1%, 30.8%, and 33.3%, respectively. The 1-year incidence of acute GVHD was 17.4%, 30.8 %, and 33.3% in the BCL, TCL, and MM groups, respectively (Fig. 3C). The 2-year incidence of chronic GVHD was 17.4%, 3.8 %, and 11.1% in the BCL, TCL, and MM groups, respectively (Fig. 3D). The characteristics of the patients with GVHD are summarized in Supplementary Table 4. No significant differences were noted in the incidence of these events between groups.

### Univariate and multivariate analyses for PFS and OS

**Fig. 5:**
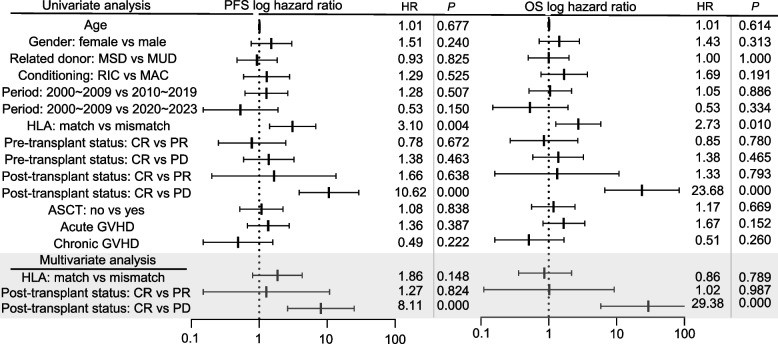
Univariate and multivariate analyses in allogeneic stem cell transplantation recipients for lymphoid malignancies. Forest plots of univariate and multivariate analyses of risk factors associated with progression-free survival and overall survival. HR, hazard ratio; RIC, reduced-intensity conditioning; MAC, myeloablative conditioning; HLA, human leukocyte antigen; ASCT, autologous stem cell transplantation; GVHD, graft-versus-host disease; NRM, non-relapse mortality

To identify factors influencing survival outcomes in patients undergoing allo-SCT, we conducted univariate and multivariate analyses of PFS and OS. Univariate analyses revealed that HLA matching between donor and recipient significantly impacted both PFS (HR: 3.10, 95% CI: 1.42–6.77, *P* = 0.004) and OS (HR: 2.73, 95% CI: 1.27–5.85, *P* = 0.037) (Fig. 5). Patients with post-transplant PD status had a significantly reduced OS rate (HR: 23.68, 95% CI: 6.69–83.74, *P* < 0.005). Other variables demonstrated no significant associations with PFS or OS. In the multivariate Cox regression model, post-transplant PD status was significantly associated with low PFS (HR: 8.11, 95% CI: 2.62–25.05, *P* < 0.005) and OS (HR: 29.38, 95% CI: 5.86–147.31, *P* < 0.005) (Fig. 5). Neither the lymphoid malignancy subtype nor the HLA match exhibited a significant association with survival in this model.

**Fig. 6:**
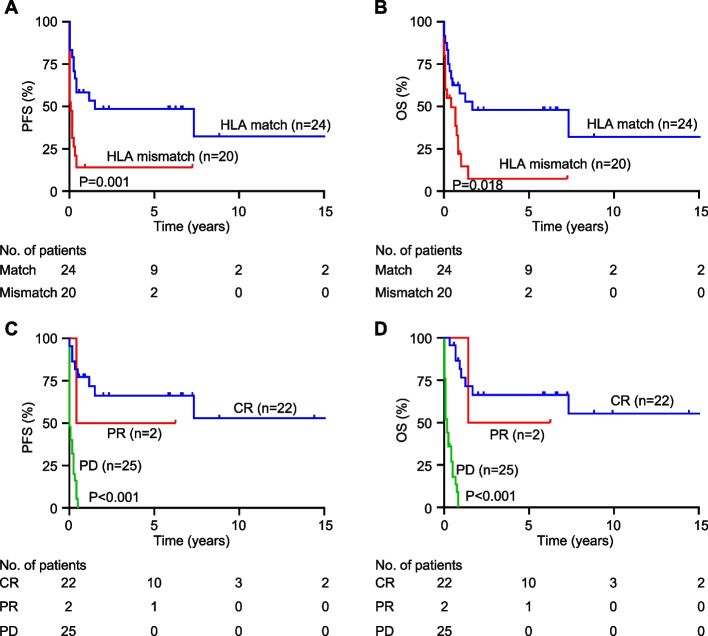
Survival outcomes according to the indicated variables in allogeneic stem cell transplantation recipients for lymphoid malignancy. **A**, **B** Progression-free survival (PFS) and overall survival (OS) of subgroups divided by human leukocyte antigen match. **C**, **D** PFS and OS of subgroups divided by status after transplant. *P*-values were determined by log-rank test

To further investigate the impact of HLA matching on survival outcomes, we compared PFS and OS between HLA-matched and mismatched patients. HLA-matched patients demonstrated significantly better PFS (*P* = 0.001) (Fig. 6A) and OS (*P* = 0.018) than those in mismatched patients (Fig. 6B). The median PFS for HLA-matched patients was 39.4 months, compared to 6.1 months for mismatched patients (Fig. 6A). The 2-year PFS probabilities were 58.3% for HLA-matched and 14.0% for HLA-mismatched patients (Fig. 6A). Median OS was 38.8 months in HLA-matched patients and 9.4 months in mismatched patients, with 2-year OS rates of 48.1% and 7.3%, respectively (Fig. 6B). Among the lymphoma subtypes, a significant association between HLA match and PFS was observed only in BCL (*P* = 0.018) (Supplementary Fig. 2). Superior OS with HLA-matched transplantation was observed only in patients with BCL (*P* < 0.001) (Supplementary Fig. 2).

In addition to HLA matching, the post-transplant status significantly influenced both PFS and OS. Patients who achieved CR (*P* < 0.001) or PR (*P* < 0.001) post-transplantation had a higher likelihood of survival than those who achieved PD (Fig. 6C, D). The median PFS for patients with CR, PR, and PD was 60.8 months, 40.0, and 1.6 months, respectively, while the median OS was 64.7 months, 46.0 months, and 3.2 months (Fig. 6C, D). The 2-year PFS rates for patients with CR and PR were 66.2% and 50.0%, respectively, with 2-year OS rates of 66.4% and 50.0%, respectively (Fig. 6C, D). None of the patients with PD had PFS or OS longer than 2 years (Fig. 6C, D). Significant associations between post-transplant status and PFS were observed in BCL (*P* < 0.001) and TCL (*P* = 0.009). Furthermore, these lymphomas demonstrated similar OS results (*P* < 0.001) (Supplementary Fig. 3).

## Discussion

Our study demonstrates that post-transplant disease status is a critical factor influencing survival outcomes in patients undergoing allo-SCT for lymphoid malignancies. Although allo-SCT is not mandatory for the subset of patients who achieve CR, our data suggest that improved outcomes can be expected. Multivariate analysis revealed that patients with PD who underwent allo-SCT had significantly shorter PFS and OS than those who achieved CR or PR, emphasizing the importance of achieving post-transplant disease control. Additionally, while HLA matching exhibited a strong association with improved PFS and OS in the univariate analysis, the technique did not retain its significance in the multivariate model. Therefore, other factors may play a more dominant role in predicting long-term outcomes. This underscores the complexity of allo-SCT and the need for individualized treatment strategies that consider multiple prognostic factors. Further research with large sample sizes is necessary to refine our understanding of these influences and improve patient selection and management for allo-SCT.

The pre-transplant status did not significantly affect survival in our study, suggesting that allo-SCT should be considered even if a patient fails to achieve CR before the procedure. Patients who achieved CR post-transplantation had significantly higher PFS and OS than those with PD, which is consistent with findings from previous studies [12]. This emphasizes the importance of achieving disease control within a short period after transplantation for superior outcomes. Although some studies have linked pre-transplant disease status to survival [19], our findings indicate that post-transplant status is a more critical factor, reinforcing the value of allo-SCT as a treatment option for patients who do not achieve CR with prior therapies.

Most HLA-mismatched patients die within 6 months of transplantation. This suggests that the survival gap between the HLA-matched and mismatched subgroups was attributed to the intensity and toxicity of the pre-transplant protocols. However, patients who underwent RIC did not have better survival rates than those who underwent MAC. In addition, HLA matching was not significantly associated with post-transplantation status. These results imply that the progression or relapse of lymphoid malignancies is responsible for reduced survival in HLA-mismatched patients. However, the possibility of treatment-related mortality (TRM) should be considered in allo-SCT. This should also be considered for pre-transplant conditioning regimens, as supported by the rapid development of RIC regimens [16, 17, 20]. In the survival curves, HLA-mismatched patients demonstrated significantly lower PFS and OS rates compared to those noted in HLA-matched patients. This indicates that although HLA-matched patients may potentially benefit from allo-SCT, treatment options other than allo-SCT should be recommended when an HLA-matched donor is not available.

Thus, in terms of eligibility criteria for allo-SCT, failure to achieve CR before transplantation should not be a barrier to this process and should not be considered an exclusion criterion. In our study, multiple patients with PD status before transplantation achieved long-term survival after undergoing allo-SCT as salvage therapy. Additionally, the availability of HLA-matched donors may indicate promising results for allo-SCT. Further studies on factors influencing status directly after transplantation can contribute to creating more elaborate criteria, as factors positively affecting post-transplantation status are likely to promote the use of allo-SCT.

The incidence of TRM was 39.1% for BCL (BCL + HD), 23.1% for TCL (TCL + NK/TCL), and 33.3% for MM, highlighting the significant risk associated with allo-SCT. These findings align with those of other studies that reported relatively high post-transplantation TRM rates [21]. Additionally, the occurrence of acute and chronic GVHD remains a major challenge, particularly affecting the skin, liver, and gut. In our study, GVHD occurred in 21 of 58 patients. Among the three patients with MM, either progression or relapse was observed in all, whereas GVHD occurred in five patients with MM. This suggests that the incidence of GVHD is not strongly associated with MM relapse. Thus, GVHD prophylaxis and management should be considered, even in the absence of disease progression or relapse. Moreover, GVHD does not necessarily increase the risk of lymphoid malignancy relapse. Previous studies have implied that the occurrence of GVHD is not significantly associated with disease progression or relapse in patients undergoing allo-SCT [22].

HLA matching had a significant effect on PFS and OS in the BCL subgroup but was not significantly associated with survival in the TCL subgroup. The fact that a high percentage of patients with BCL underwent ASCT before undergoing allo-SCT may explain this difference. The process of debulking malignancies using ASCT intensifies the GVL effect [23]. Although numerous studies have demonstrated the effect of ASCT in patients with BCL, limited evidence exists on the relationship between ASCT and TCL, which may explain the widespread use of ASCT in BCL [24, 25].

Although novel cellular therapies, such as CAR-T, display promising results, allo-SCT remains a strong option, particularly considering the advantages it offers in HLA-matched transplants and patients achieving CR or PR post-transplantation. These factors are associated with improved survival outcomes. Further studies with large sample sizes are needed to refine our understanding of the prognostic factors and outcomes associated with allo-SCT, thereby enabling accurate selection and treatment strategies for patients with lymphoid malignancies.

## Supplementary Information

Supplementary Material 1.

Supplementary Material 2.

## Data Availability

No datasets were generated or analysed during the current study.
