# Supplementary material for: Prognostic factors and treatment outcomes of allogeneic stem cell transplantation in lymphoid malignancy
Source: Blood Res. 2025 Feb 10;60(1):12. doi: 10.1007/s44313-025-00060-y (PMC11811309; doi:10.1007/s44313-025-00060-y)
Supplement: Supplementary file 1 — Supplementary Material 1. [file 44313_2025_60_MOESM1_ESM.pptx]

## Slide 1
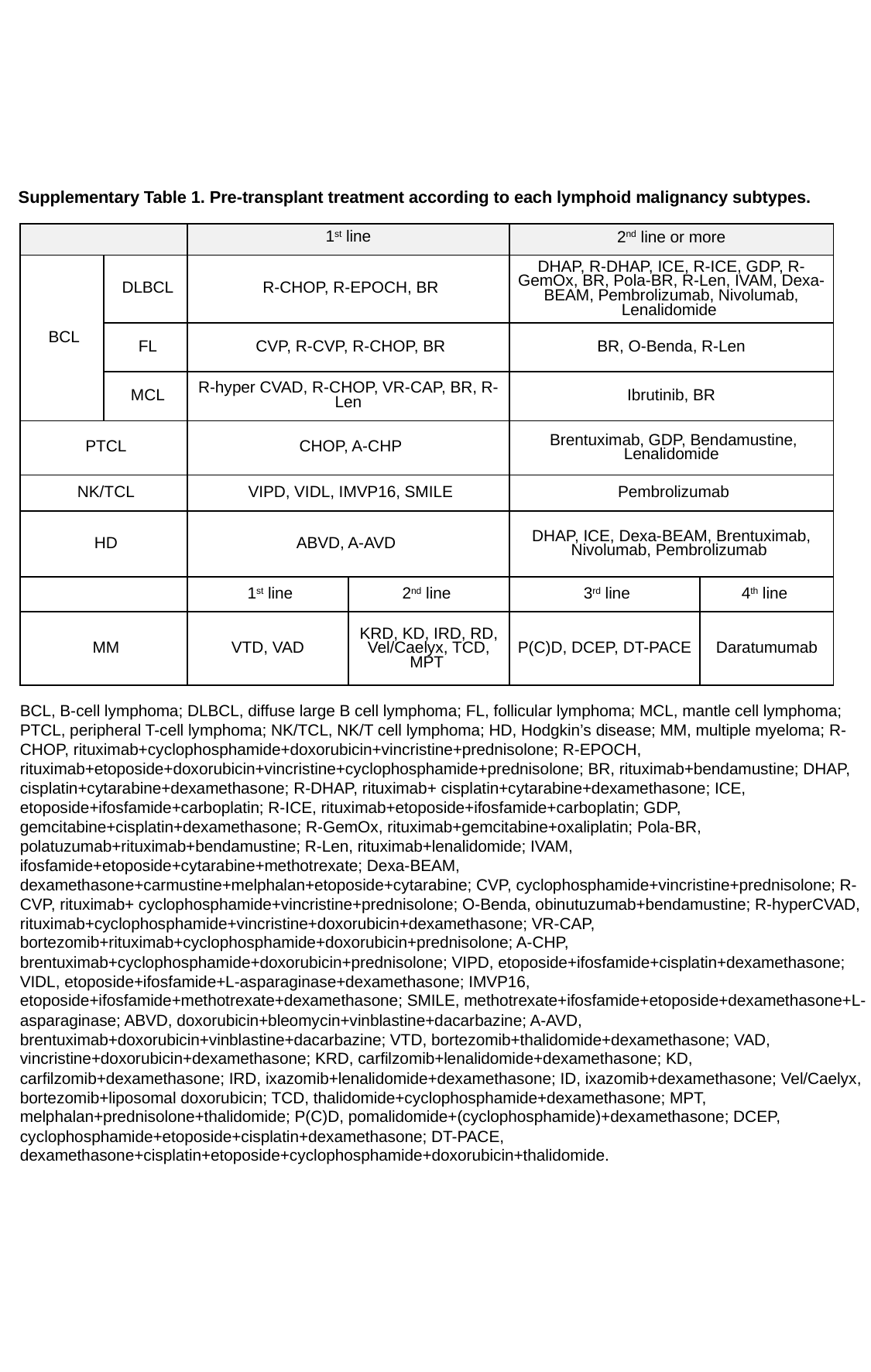

Supplementary Table 1. Pre-transplant treatment according to each lymphoid malignancy subtypes.
| | | 1st line | | 2nd line or more | |
| --- | --- | --- | --- | --- | --- |
| BCL | DLBCL | R-CHOP, R-EPOCH, BR | | DHAP, R-DHAP, ICE, R-ICE, GDP, R-GemOx, BR, Pola-BR, R-Len, IVAM, Dexa-BEAM, Pembrolizumab, Nivolumab, Lenalidomide | |
| | FL | CVP, R-CVP, R-CHOP, BR | | BR, O-Benda, R-Len | |
| | MCL | R-hyper CVAD, R-CHOP, VR-CAP, BR, R-Len | | Ibrutinib, BR | |
| PTCL | | CHOP, A-CHP | | Brentuximab, GDP, Bendamustine, Lenalidomide | |
| NK/TCL | | VIPD, VIDL, IMVP16, SMILE | | Pembrolizumab | |
| HD | | ABVD, A-AVD | | DHAP, ICE, Dexa-BEAM, Brentuximab, Nivolumab, Pembrolizumab | |
| | | 1st line | 2nd line | 3rd line | 4th line |
| MM | | VTD, VAD | KRD, KD, IRD, RD, Vel/Caelyx, TCD, MPT | P(C)D, DCEP, DT-PACE | Daratumumab |
BCL, B-cell lymphoma; DLBCL, diffuse large B cell lymphoma; FL, follicular lymphoma; MCL, mantle cell lymphoma; PTCL, peripheral T-cell lymphoma; NK/TCL, NK/T cell lymphoma; HD, Hodgkin’s disease; MM, multiple myeloma; R-CHOP, rituximab+cyclophosphamide+doxorubicin+vincristine+prednisolone; R-EPOCH, rituximab+etoposide+doxorubicin+vincristine+cyclophosphamide+prednisolone; BR, rituximab+bendamustine; DHAP, cisplatin+cytarabine+dexamethasone; R-DHAP, rituximab+ cisplatin+cytarabine+dexamethasone; ICE, etoposide+ifosfamide+carboplatin; R-ICE, rituximab+etoposide+ifosfamide+carboplatin; GDP, gemcitabine+cisplatin+dexamethasone; R-GemOx, rituximab+gemcitabine+oxaliplatin; Pola-BR, polatuzumab+rituximab+bendamustine; R-Len, rituximab+lenalidomide; IVAM, ifosfamide+etoposide+cytarabine+methotrexate; Dexa-BEAM, dexamethasone+carmustine+melphalan+etoposide+cytarabine; CVP, cyclophosphamide+vincristine+prednisolone; R-CVP, rituximab+ cyclophosphamide+vincristine+prednisolone; O-Benda, obinutuzumab+bendamustine; R-hyperCVAD, rituximab+cyclophosphamide+vincristine+doxorubicin+dexamethasone; VR-CAP, bortezomib+rituximab+cyclophosphamide+doxorubicin+prednisolone; A-CHP, brentuximab+cyclophosphamide+doxorubicin+prednisolone; VIPD, etoposide+ifosfamide+cisplatin+dexamethasone; VIDL, etoposide+ifosfamide+L-asparaginase+dexamethasone; IMVP16, etoposide+ifosfamide+methotrexate+dexamethasone; SMILE, methotrexate+ifosfamide+etoposide+dexamethasone+L-asparaginase; ABVD, doxorubicin+bleomycin+vinblastine+dacarbazine; A-AVD, brentuximab+doxorubicin+vinblastine+dacarbazine; VTD, bortezomib+thalidomide+dexamethasone; VAD, vincristine+doxorubicin+dexamethasone; KRD, carfilzomib+lenalidomide+dexamethasone; KD, carfilzomib+dexamethasone; IRD, ixazomib+lenalidomide+dexamethasone; ID, ixazomib+dexamethasone; Vel/Caelyx, bortezomib+liposomal doxorubicin; TCD, thalidomide+cyclophosphamide+dexamethasone; MPT, melphalan+prednisolone+thalidomide; P(C)D, pomalidomide+(cyclophosphamide)+dexamethasone; DCEP, cyclophosphamide+etoposide+cisplatin+dexamethasone; DT-PACE, dexamethasone+cisplatin+etoposide+cyclophosphamide+doxorubicin+thalidomide.

## Slide 2
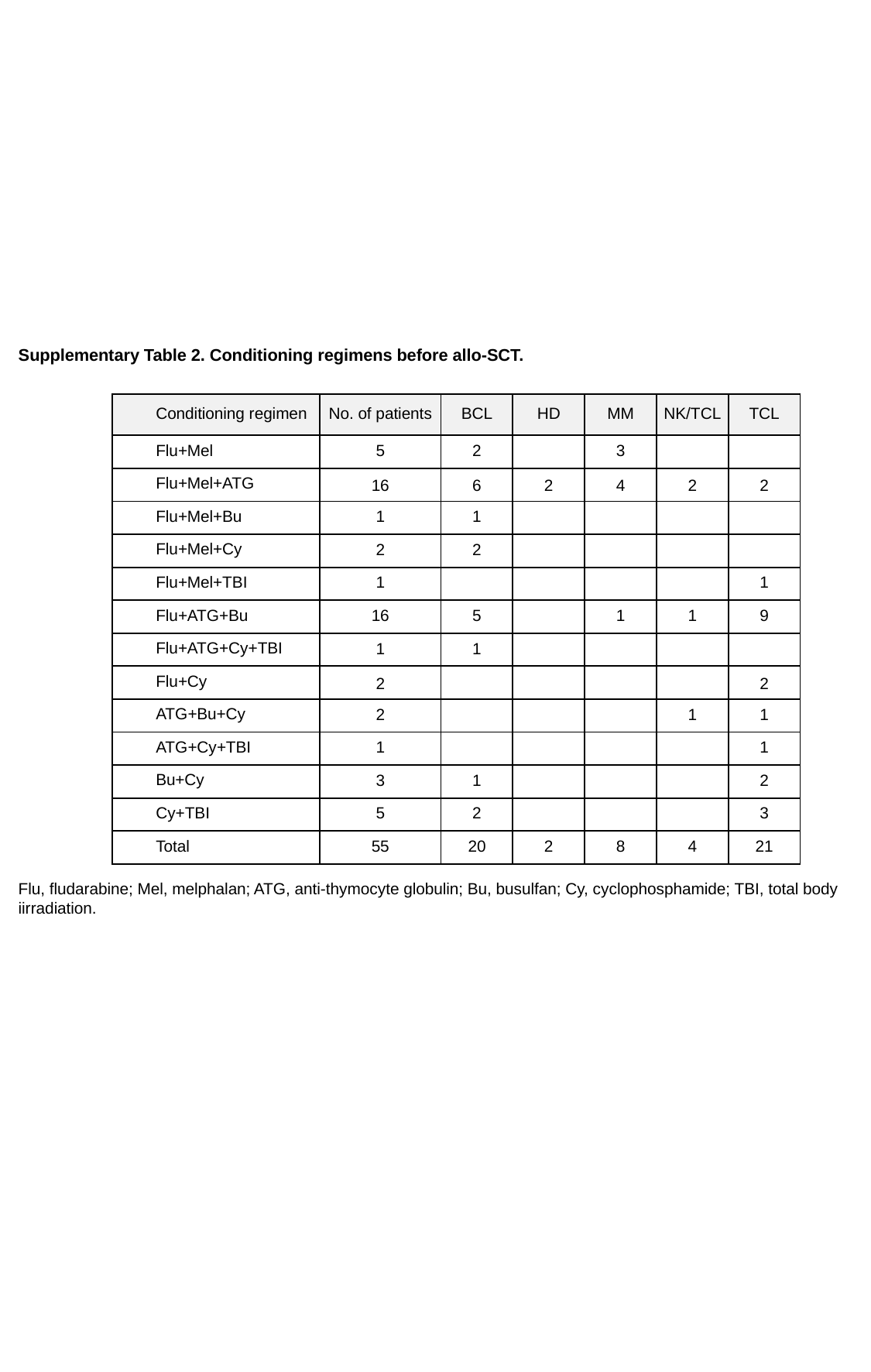

Supplementary Table 2. Conditioning regimens before allo-SCT.
| Conditioning regimen | No. of patients | BCL | HD | MM | NK/TCL | TCL |
| --- | --- | --- | --- | --- | --- | --- |
| Flu+Mel | 5 | 2 | | 3 | | |
| Flu+Mel+ATG | 16 | 6 | 2 | 4 | 2 | 2 |
| Flu+Mel+Bu | 1 | 1 | | | | |
| Flu+Mel+Cy | 2 | 2 | | | | |
| Flu+Mel+TBI | 1 | | | | | 1 |
| Flu+ATG+Bu | 16 | 5 | | 1 | 1 | 9 |
| Flu+ATG+Cy+TBI | 1 | 1 | | | | |
| Flu+Cy | 2 | | | | | 2 |
| ATG+Bu+Cy | 2 | | | | 1 | 1 |
| ATG+Cy+TBI | 1 | | | | | 1 |
| Bu+Cy | 3 | 1 | | | | 2 |
| Cy+TBI | 5 | 2 | | | | 3 |
| Total | 55 | 20 | 2 | 8 | 4 | 21 |
Flu, fludarabine; Mel, melphalan; ATG, anti-thymocyte globulin; Bu, busulfan; Cy, cyclophosphamide; TBI, total body iirradiation.

## Slide 3
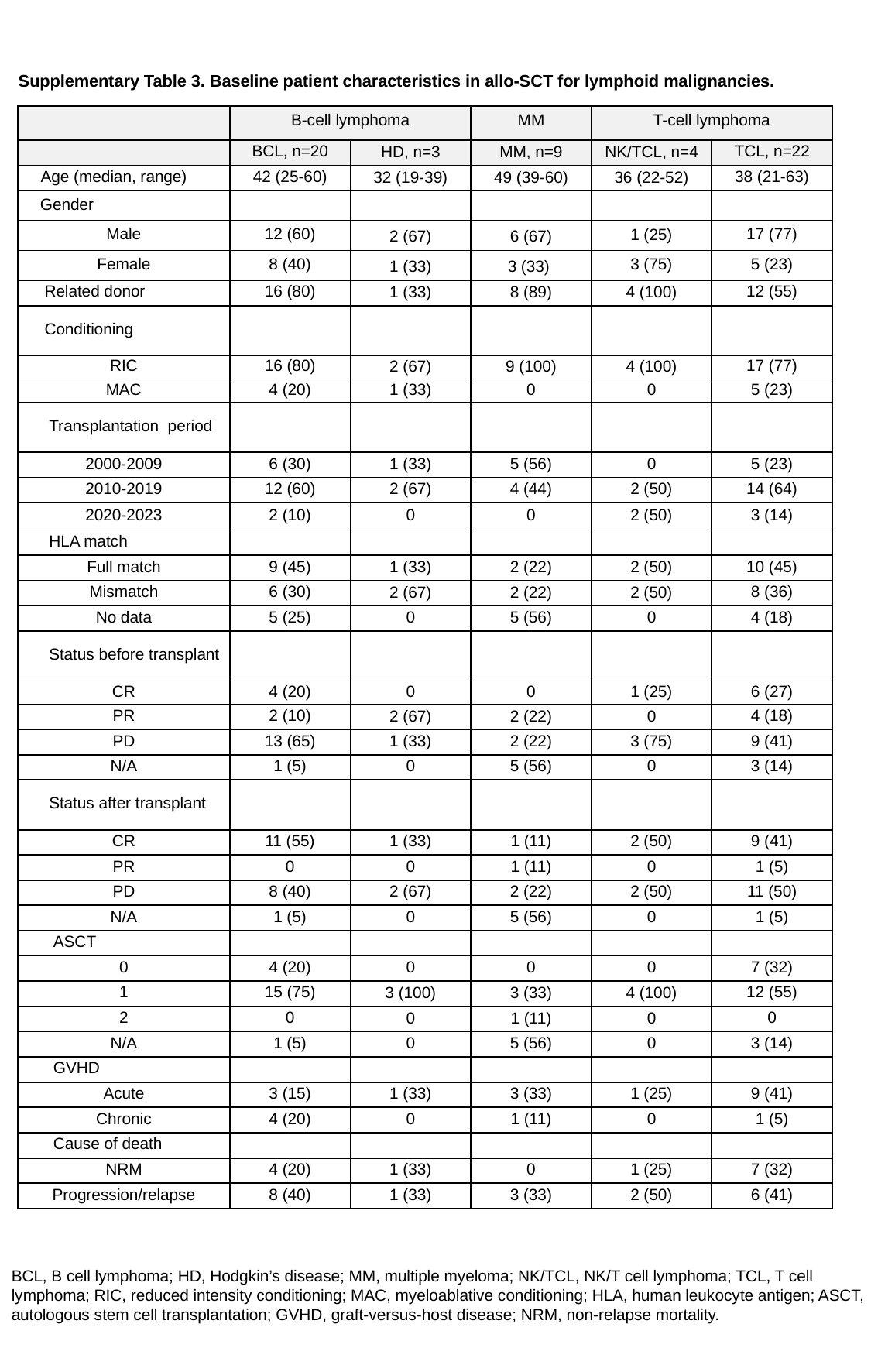

Supplementary Table 3. Baseline patient characteristics in allo-SCT for lymphoid malignancies.
| | B-cell lymphoma | | MM | T-cell lymphoma | |
| --- | --- | --- | --- | --- | --- |
| | BCL, n=20 | HD, n=3 | MM, n=9 | NK/TCL, n=4 | TCL, n=22 |
| Age (median, range) | 42 (25-60) | 32 (19-39) | 49 (39-60) | 36 (22-52) | 38 (21-63) |
| Gender | | | | | |
| Male | 12 (60) | 2 (67) | 6 (67) | 1 (25) | 17 (77) |
| Female | 8 (40) | 1 (33) | 3 (33) | 3 (75) | 5 (23) |
| Related donor | 16 (80) | 1 (33) | 8 (89) | 4 (100) | 12 (55) |
| Conditioning | | | | | |
| RIC | 16 (80) | 2 (67) | 9 (100) | 4 (100) | 17 (77) |
| MAC | 4 (20) | 1 (33) | 0 | 0 | 5 (23) |
| Transplantation period | | | | | |
| 2000-2009 | 6 (30) | 1 (33) | 5 (56) | 0 | 5 (23) |
| 2010-2019 | 12 (60) | 2 (67) | 4 (44) | 2 (50) | 14 (64) |
| 2020-2023 | 2 (10) | 0 | 0 | 2 (50) | 3 (14) |
| HLA match | | | | | |
| Full match | 9 (45) | 1 (33) | 2 (22) | 2 (50) | 10 (45) |
| Mismatch | 6 (30) | 2 (67) | 2 (22) | 2 (50) | 8 (36) |
| No data | 5 (25) | 0 | 5 (56) | 0 | 4 (18) |
| Status before transplant | | | | | |
| CR | 4 (20) | 0 | 0 | 1 (25) | 6 (27) |
| PR | 2 (10) | 2 (67) | 2 (22) | 0 | 4 (18) |
| PD | 13 (65) | 1 (33) | 2 (22) | 3 (75) | 9 (41) |
| N/A | 1 (5) | 0 | 5 (56) | 0 | 3 (14) |
| Status after transplant | | | | | |
| CR | 11 (55) | 1 (33) | 1 (11) | 2 (50) | 9 (41) |
| PR | 0 | 0 | 1 (11) | 0 | 1 (5) |
| PD | 8 (40) | 2 (67) | 2 (22) | 2 (50) | 11 (50) |
| N/A | 1 (5) | 0 | 5 (56) | 0 | 1 (5) |
| ASCT | | | | | |
| 0 | 4 (20) | 0 | 0 | 0 | 7 (32) |
| 1 | 15 (75) | 3 (100) | 3 (33) | 4 (100) | 12 (55) |
| 2 | 0 | 0 | 1 (11) | 0 | 0 |
| N/A | 1 (5) | 0 | 5 (56) | 0 | 3 (14) |
| GVHD | | | | | |
| Acute | 3 (15) | 1 (33) | 3 (33) | 1 (25) | 9 (41) |
| Chronic | 4 (20) | 0 | 1 (11) | 0 | 1 (5) |
| Cause of death | | | | | |
| NRM | 4 (20) | 1 (33) | 0 | 1 (25) | 7 (32) |
| Progression/relapse | 8 (40) | 1 (33) | 3 (33) | 2 (50) | 6 (41) |
BCL, B cell lymphoma; HD, Hodgkin’s disease; MM, multiple myeloma; NK/TCL, NK/T cell lymphoma; TCL, T cell lymphoma; RIC, reduced intensity conditioning; MAC, myeloablative conditioning; HLA, human leukocyte antigen; ASCT, autologous stem cell transplantation; GVHD, graft-versus-host disease; NRM, non-relapse mortality.

## Slide 4
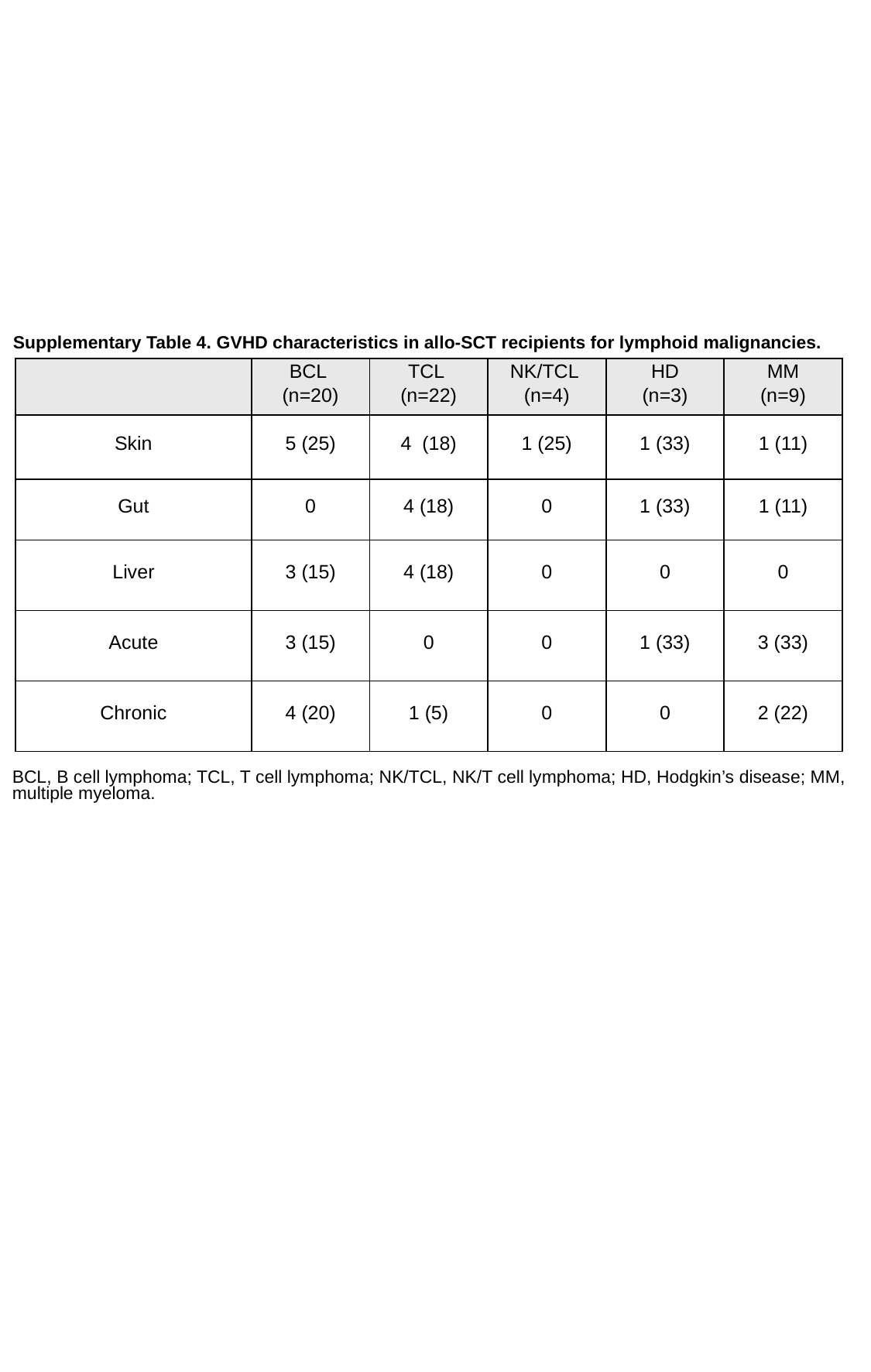

Supplementary Table 4. GVHD characteristics in allo-SCT recipients for lymphoid malignancies.
| | BCL (n=20) | TCL (n=22) | NK/TCL (n=4) | HD (n=3) | MM (n=9) |
| --- | --- | --- | --- | --- | --- |
| Skin | 5 (25) | 4 (18) | 1 (25) | 1 (33) | 1 (11) |
| Gut | 0 | 4 (18) | 0 | 1 (33) | 1 (11) |
| Liver | 3 (15) | 4 (18) | 0 | 0 | 0 |
| Acute | 3 (15) | 0 | 0 | 1 (33) | 3 (33) |
| Chronic | 4 (20) | 1 (5) | 0 | 0 | 2 (22) |
BCL, B cell lymphoma; TCL, T cell lymphoma; NK/TCL, NK/T cell lymphoma; HD, Hodgkin’s disease; MM, multiple myeloma.
