# Supplementary material for: Prognostic factors and treatment outcomes of allogeneic stem cell transplantation in lymphoid malignancy
Source: Blood Res. 2025 Feb 10;60(1):12. doi: 10.1007/s44313-025-00060-y (PMC11811309; doi:10.1007/s44313-025-00060-y)
Supplement: Supplementary file 2 — Supplementary Material 2. [file 44313_2025_60_MOESM2_ESM.pptx]

## Slide 1
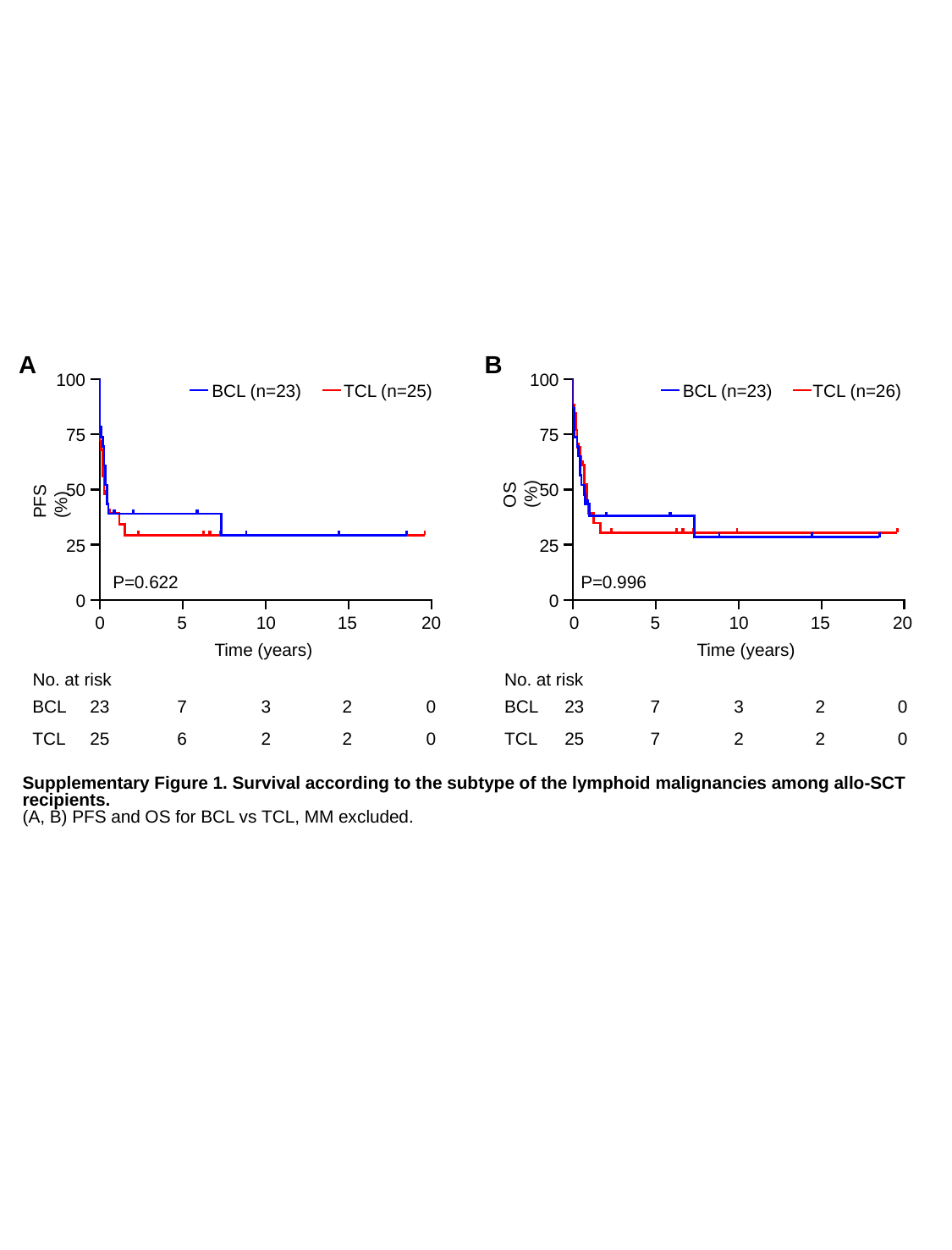

A
B
100
100
BCL (n=23)
TCL (n=25)
BCL (n=23)
TCL (n=26)
75
75
PFS (%)
OS (%)
50
50
25
25
P=0.622
P=0.996
0
0
0
5
10
15
20
0
5
10
15
20
Time (years)
Time (years)
No. at risk
No. at risk
BCL
23
7
3
2
0
BCL
23
7
3
2
0
TCL
25
6
2
2
0
TCL
25
7
2
2
0
Supplementary Figure 1. Survival according to the subtype of the lymphoid malignancies among allo-SCT recipients.
(A, B) PFS and OS for BCL vs TCL, MM excluded.

## Slide 2
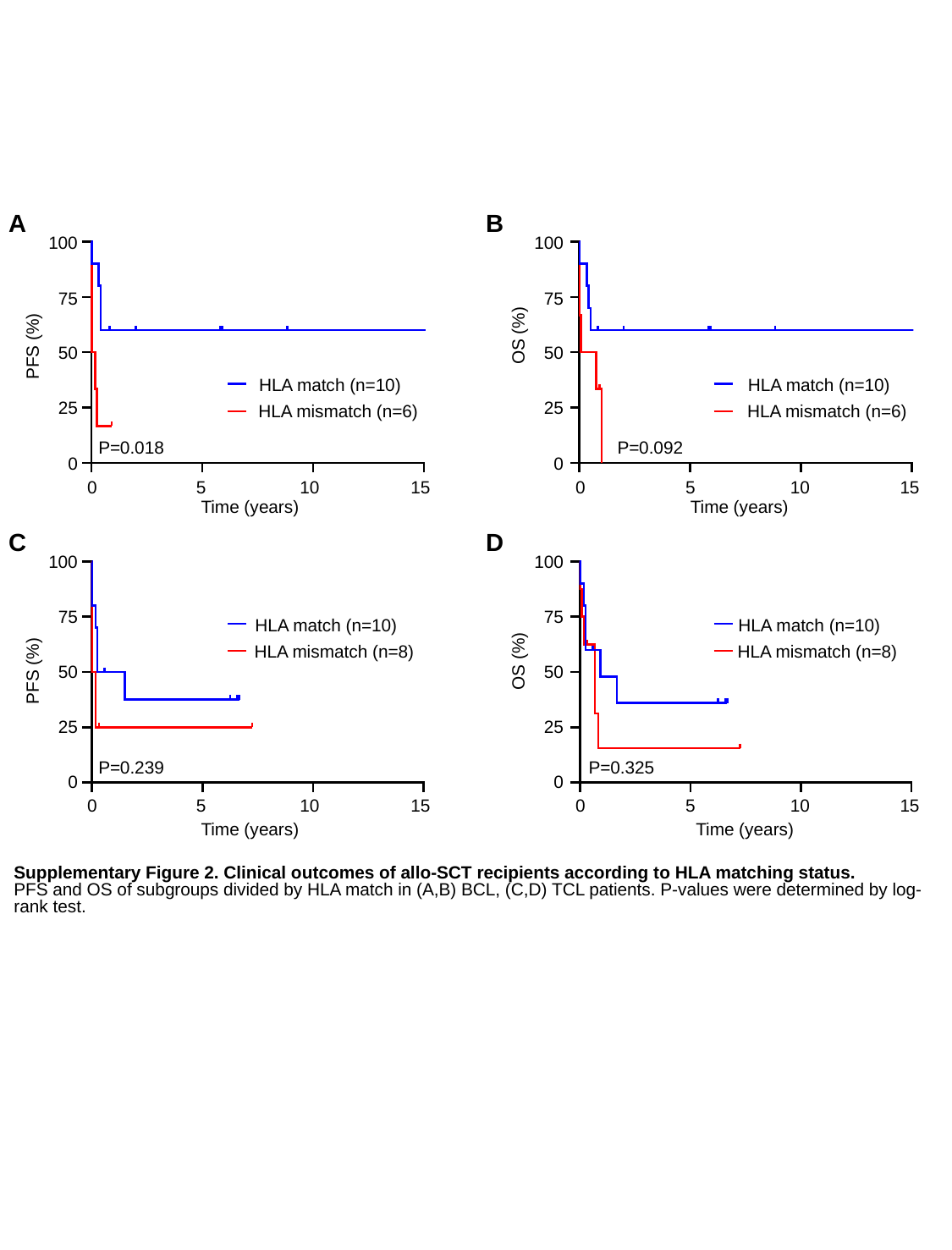

OS (%)
A
B
PFS (%)
100
100
75
75
50
50
HLA match (n=10)
HLA match (n=10)
HLA mismatch (n=6)
HLA mismatch (n=6)
25
25
P=0.018
P=0.092
0
0
0
5
10
15
0
5
10
15
Time (years)
Time (years)
OS (%)
C
D
PFS (%)
100
100
75
75
HLA match (n=10)
HLA match (n=10)
HLA mismatch (n=8)
HLA mismatch (n=8)
50
50
25
25
P=0.239
P=0.325
0
0
0
5
10
15
0
5
10
15
Time (years)
Time (years)
Supplementary Figure 2. Clinical outcomes of allo-SCT recipients according to HLA matching status.
PFS and OS of subgroups divided by HLA match in (A,B) BCL, (C,D) TCL patients. P-values were determined by log-rank test.

## Slide 3
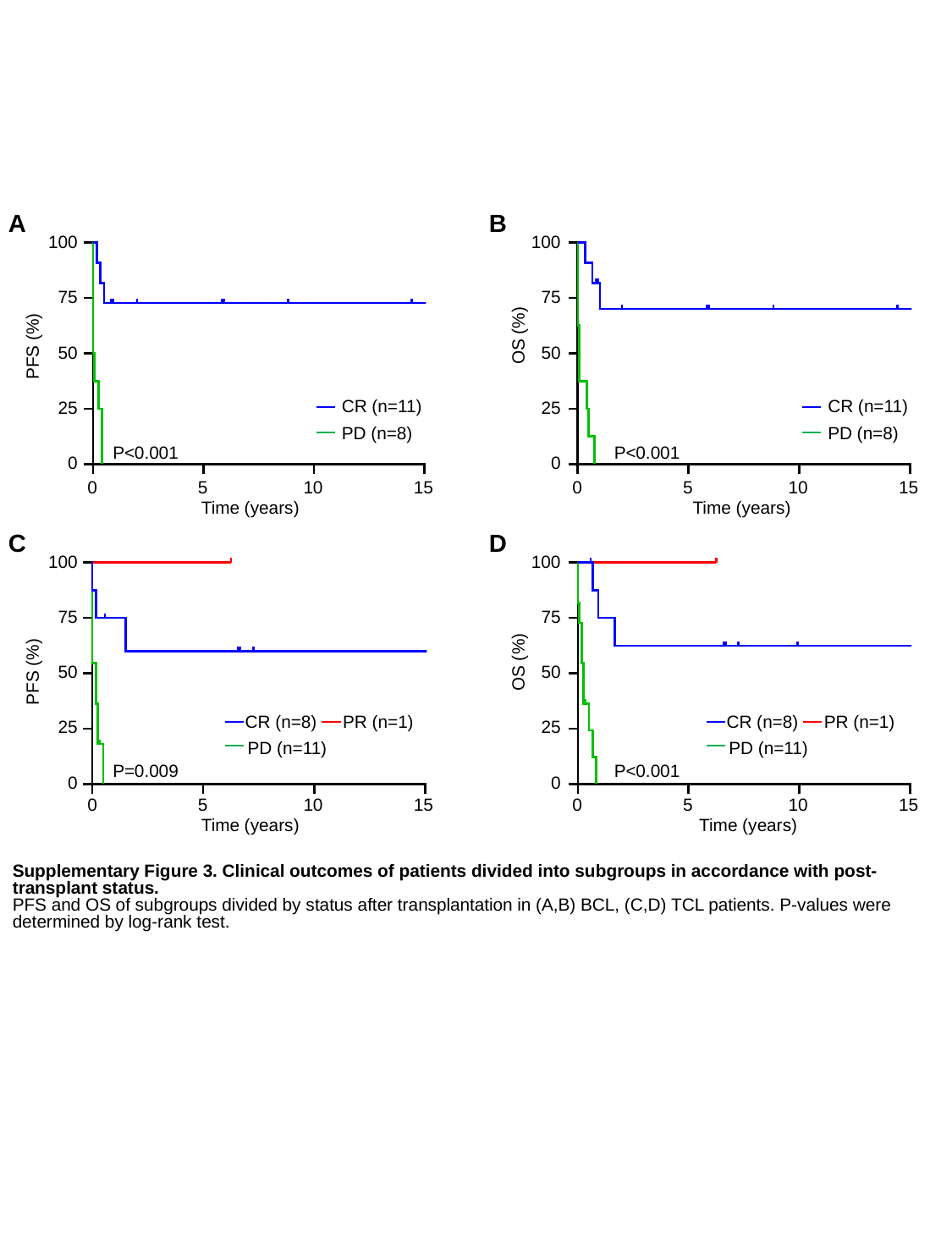

OS (%)
A
B
PFS (%)
100
100
75
75
50
50
CR (n=11)
CR (n=11)
25
25
PD (n=8)
PD (n=8)
P<0.001
P<0.001
0
0
0
5
10
15
0
5
10
15
Time (years)
Time (years)
OS (%)
C
D
PFS (%)
100
100
75
75
50
50
CR (n=8)
PR (n=1)
CR (n=8)
PR (n=1)
25
25
PD (n=11)
PD (n=11)
P=0.009
P<0.001
0
0
0
5
10
15
0
5
10
15
Time (years)
Time (years)
Supplementary Figure 3. Clinical outcomes of patients divided into subgroups in accordance with post-transplant status.
PFS and OS of subgroups divided by status after transplantation in (A,B) BCL, (C,D) TCL patients. P-values were determined by log-rank test.
